# Respiratory motion correction in F-18-FDG PET/CT impacts lymph node assessment in lung cancer patients

**DOI:** 10.1186/s13550-022-00926-7

**Published:** 2022-09-15

**Authors:** Benjamin Noto, Wolfgang Roll, Laura Zinken, Robert Rischen, Laura Kerschke, Georg Evers, Walter Heindel, Michael Schäfers, Florian Büther

**Affiliations:** 1 Department of Nuclear Medicine, University Hospital Münster, Münster, Germany; 2 Clinical for Radiology, University Hospital Münster, Albert-Schweitzer-Campus 1, 48149 Münster, Germany; 3 Institute of Biostatistics and Clinical Research, University of Münster, Münster, Germany; 4 Department of Medicine A, Hematology, Oncology and Pulmonary Medicine, University Hospital Münster, Münster, Germany; 5 European Institute for Molecular Imaging, University of Münster, Münster, Germany; 6 West German Cancer Centre (WTZ), Münster, Germany

**Keywords:** PET, PET/CT, Motion correction, Lung cancer, Staging

## Abstract

**Backgrounds:**

Elastic motion correction in PET has been shown to increase image quality and quantitative measurements of PET datasets affected by respiratory motion. However, little is known on the impact of respiratory motion correction on clinical image evaluation in oncologic PET. This study evaluated the impact of motion correction on expert readers’ lymph node assessment of lung cancer patients.

**Methods:**

Forty-three patients undergoing F-18-FDG PET/CT for the staging of suspected lung cancer were included. Three different PET reconstructions were investigated: non-motion-corrected (“static”), belt gating-based motion-corrected (“BG-MC”) and data-driven gating-based motion-corrected (“DDG-MC”). Assessment was conducted independently by two nuclear medicine specialists blinded to the reconstruction method on a six-point scale $s$ ranging from “certainly negative” (1) to “certainly positive” (6). Differences in $s$ between reconstruction methods, accounting for variation caused by readers, were assessed by nonparametric regression analysis of longitudinal data. From $s$, a dichotomous score for N1, N2, and N3 (“negative,” “positive”) and a subjective certainty score were derived. SUV and metabolic tumor volumes (MTV) were compared between reconstruction methods.

**Results:**

BG-MC resulted in higher scores for N1 compared to static (*p* = 0.001), whereas DDG-MC resulted in higher scores for N2 compared to static (*p* = 0.016). Motion correction resulted in the migration of N1 from tumor free to metastatic on the dichotomized score, consensually for both readers, in 3/43 cases and in 2 cases for N2. SUV was significantly higher for motion-corrected PET, while MTV was significantly lower (all *p* < 0.003). No significant differences in the certainty scores were noted.

**Conclusions:**

PET motion correction resulted in significantly higher lymph node assessment scores of expert readers. Significant effects on quantitative PET parameters were seen; however, subjective reader certainty was not improved.

## Introduction

Lung cancer is one of the most common cancers and the leading cause of cancer-related deaths worldwide [1]. F-18-FDG PET/CT is implemented in the initial staging of lung cancer patients, especially for the assessment of lymph node involvement and exclusion of distant metastases [2, 3]. Moreover, its use is recommended for the assessment of suspicious pulmonary nodules [4]. Sensitivity of F-18-FDG PET/CT is high for distinguishing malignant from benign solitary pulmonary nodules; however, it demonstrated low specificity [5]. Additionally, sensitivity of F-18-FDG PET/CT is limited in the evaluation of lymph nodes [6, 7]. Thus decisions on management in lung cancer patients should not be based on F-18-FDG PET/CT alone, and improvements in lymph node assessment are warranted [6].

Respiratory motion is a well-known source of image artifacts and erroneous quantification in thoracic and abdominal PET, resulting in decreased apparent tracer uptake quantification, increased MTV, and losses in effective spatial resolution [8–10]. To overcome this, a wide range of motion correction algorithms for PET have been introduced and investigated during the last two decades, with the most practical and robust ones now becoming established in clinical scans (albeit at a slow rate). Historically, the proposed methods range from comparatively simple approaches avoiding respiratory motion effects by prolonged scanning of a defined respiratory phase (most often end-expiration) [11] to more advanced solutions comprising gated reconstructions where an additionally acquired signal representing the respiratory phase of a patient during the scan is used to reconstruct only coincidence events emitted during a specified respiratory state [12, 13]. An important subset of the latter methods, software or data-driven gating (DDG) is based on analyzing measured PET raw data to calculate breathing signals instead of using additional hardware to record these signals, thus potentially simplifying clinical scans and increasing patient comfort [14–17]. Finally, fully motion-corrected reconstructions have been recently introduced by taking all measured PET data into account, rather than just a subset determined by a specified gating approach [18, 19].

Clinical studies already demonstrated that gated or motion-corrected PET reconstructions typically resulted in higher tracer uptake values, smaller lesion volumes and subjectively “sharper” images [10, 20, 21]. Few studies have investigated the role of PET-derived gating on diagnostic accuracy for the detection and characterization of suspicious solitary pulmonary nodules [22, 23]. However, besides these basic, directly image-derived parameters and first clinical applications, not much is known about the impact of motion-corrected PET on staging and clinical decisions making. First results of a multi-tracer study indicate that DDG might result in changes in clinical PET reports and might even change further clinical management in many different types of cancer [24]. The authors strongly encourage dedicated future studies in different disease settings [24].

We therefore opted to investigate the impact of fully motion-corrected PET reconstructions, based on both hardware- and software-derived gating, in F-18-FDG PET/CT staging scans of lung cancer patients. In particular, we were interested in the subjective differences in lymph node assessment of expert readers using non-motion-corrected PET and motion-corrected PET, respectively.

## Materials and methods

### Patient data

In this retrospective analysis datasets of 43 patients who underwent initial F-18-FDG PET/CT for staging of suspected lung cancer at our facility between December 2018 and December 2020 were included. Patients with prior resection of the primary tumor were excluded. The study design was approved by the local ethics committee of the University of Münster (AZ 2019-024-f-S, 2021-172-f-S), and was performed in accordance with the 1964 Helsinki declaration and its later amendments. The need for written informed consent was waived due to the retrospective nature of the study.

### PET/CT scans

The patients fasted overnight before the PET/CT scan. They received 3 MBq/kg body mass of F-18-FDG i.v. approximately one hour prior to the scan which was performed on a Biograph mCT (Siemens Healthcare GmbH, Erlangen, Germany) capable of time-of-flight and continuous bed motion (axial PET field-of-view, 21.8 cm; spatial resolution at center, 4 mm full width at half maximum; sinogram sizes, 400 × 168; time-of-flight bins, 13) [25]. Patients were scanned in a supine position with the arms above the head. During the examination, the respiratory gating system AZ-733 V (Anzai Co., Tokyo, Japan) recorded respiratory signals for subsequent gating (belt gating, BG) and motion correction.

Scanning ranges were from the head or neck down to the proximal femur. End-expiratory low-dose CT scans were performed (tube voltage, 120 kV; effective current, 18 mAs; slice thickness, 3.0 mm; duration, 10–20 s) followed by PET in continuous bed motion (free breathing; speed, 1.1 mm/s; duration, 500–900 s).

### Reconstructions and motion correction

**Fig. 1:**
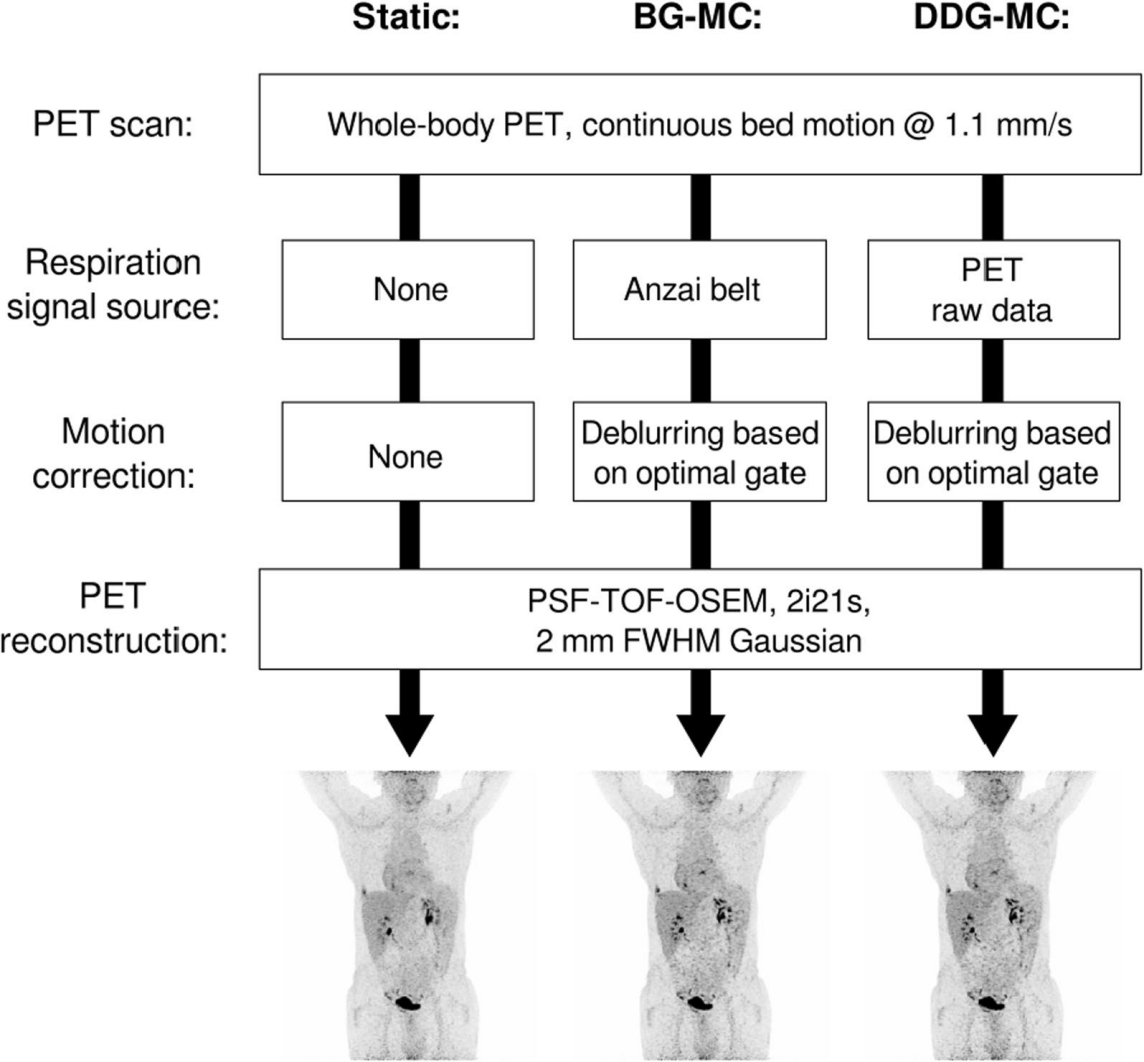
Reconstruction workflow used for the three PET images (“static,” “BG-MC” and “DDG-MC”) performed within this study

Three different PET reconstructions were investigated within this study (Fig. 1): (1) Static reconstruction without motion correction (“static”); (2) elastic motion-corrected reconstruction based on the belt gating signal (“BG-MC”); and (3) elastic motion-corrected reconstruction based on PET raw data-driven gating signal (“DDG-MC”).

The applied DDG algorithm is based on a spectral analysis of continuous bed motion PET raw data and is described in detail elsewhere [17, 21]. Briefly, it divides the raw data into axial regions of 80 mm length, where measured events are back-projected into the most likely origin voxel according to their time-of-flight bin. The predominant respiratory frequency was then identified by the maximum in the power spectrum of the standard deviation along the anterior–posterior axis over time. Voxels that demonstrated fluctuations close to this frequency were then used to define a mask of regions affected by respiration. Respiratory signals for each axial region were then calculated by phase- and mask-weighted summation of voxel time–activity curves and finally concatenated and normalized to give an overall DDG signal for the whole PET scan.

Signals from both sources were used for elastic motion-corrected PET reconstructions by first reconstructing the “optimal gate” comprising coincidence data from the narrowest signal amplitude interval covering 35% of the total data, giving a good compromise between motion resolution and data statistics, and then using mass-preserving optical flow techniques to determine a motion vector field between the gated and a static reconstruction. This vector field was then finally used in an effective deblurring step within a motion-corrected image reconstruction [18, 19], resulting in BG-MC and DDG-MC datasets.

All reconstructions were based on an ordinary Poisson ordered subset expectation maximization (2 iterations, 21 subsets, 2 mm full width at half maximum Gaussian post-reconstruction filter, 400 × 400 image matrix, 2.04 × 2.04 × 2.03 mm^3^ voxel volume; e7 toolbox, Siemens Healthcare GmbH, Erlangen, Germany) with point-spread-function and time-of-flight data, normalization, and random correction; attenuation and scatter correction were based on the measured CT data. Overall, three PET and one CT image dataset per patient were thus subsequently analyzed.

### Image Assessment

All PET and CT images were anonymized and sent to a syngo.via workstation (Oncology tool, Siemens Healthcare GmbH, Erlangen, Germany) where they were presented independently to two nuclear medicine specialists (BN, WR) with more than five years of experience in PET/CT imaging. One of the three PET reconstructions, the CT image and a fused PET-CT image were made available to a reader. The three different PET reconstructions (static, BG-MC, DDG-MC) for any given scan were presented in random order and in different sessions in an interval of at least 2 weeks to reduce bias. The readers were blinded for the actual type of reconstruction.

The lymph node (N) and distant metastasis (M) status was assessed, with the N rating further divided into the three different lymph node regions N1 (ipsilateral peribronchial and/or hilar lymph nodes), N2 (ipsilateral mediastinal and/or subcarinal lymph nodes), and N3 (contralateral mediastinal and/or hilar, as well as any supraclavicular lymph nodes), following the TNM staging system for lung cancer of the American Joint Commission of Cancer (AJCC) and the Union Internationale Contre la Cancer (UICC) [26]. For every reconstruction, these three N regions and the M status were independently rated on an ordinal scale $s$ ranging from 1 (“certainly negative”), 2 (“probably negative”), 3 (“doubtfully negative”), 4 (“doubtfully positive”), 5 (“probably positive”), to 6 (“certainly positive”). Derived from this score, a simplified dichotomous score $d$ was defined as 0 for negative findings (scale values of 1, 2, 3) and 1 for positive findings (scale values of 4, 5, 6).

Finally, to quantify the subjective certainty of the readers, an ordinal certainty score was calculated as

$$c = \left| {3.5 - s} \right| + 0.5$$

with 1 denoting least certainty and 3 denoting highest certainty.

Additionally, the primary tumor and the most prominent lymph nodes visible in each region N1, N2 and N3 were characterized by their standardized uptake values SUV_max_, and SUV_mean_, and the metabolic tumor volume (MTV) in each reconstruction.

### Statistical analysis

Analyses were performed using R statistical software version 3.6.1 (The R Foundation, r-project.org). All reported *p* values are two-sided. Normally distributed data were described using mean and standard deviation. Non-normally distributed data were described using median and interquartile range. Normality was assessed by analysis of histograms and skewness statistics.

Interobserver agreement for TNM staging using the ordinal scale $s$ was assessed using Cohen’s weighted kappa statistics. In the primary statistical analysis differences in the ordinal score values $s$ between reconstruction methods were assessed for each region by nonparametric analysis of longitudinal data in factorial experiments using the R package nparLD [27], as were differences in the certainty score $c$. The method accounts for dependencies between measurements on the same patient (i.e., for a given region each patient provides a measurement per reconstruction method and reader, resulting in six observations per patient). A multiple comparison procedure based on the closed testing principle [28] was applied to each region using a (multiple) significance level of 0.05 per region. Following this principle, a single pairwise comparison was considered significant, if both the overall comparison and the pairwise comparison resulted in a p value ≤ 0.05.

SUV and MTV showed a non-normal distribution in histograms analysis. Differences in SUV and volumes between methods were assessed in an exploratory analysis using Friedman’s test. Wilcoxon signed-rank tests were applied as post hoc procedure. *p* values ≤ 0.05 were considered significant.

## Results

### Patient characteristics

Forty-three patients with a median age of 70 years (15 women, 28 men) were included in this retrospective analysis. For further patients’ characteristics, see Table 1.

**Table 1:** Patients’ characteristics

Category	n/median	Percentage/range
Subjects	43	
Age [years]	70	(47–85)
Female	15	34.9%
Male	28	65.1%
Histology		
Non-small cell lung cancer	29	67.4%
Small cell lung cancer	3	7.0%
Unknown	11	25.6%

### Interreader agreement

Interreader agreement for score $s$ was excellent for all locations and image reconstructions, according to the magnitude guidelines as suggested by Landis and Koch [29], with weighted kappa values ranging from 0.88 to 0.96 (Table 2).

**Table 2:** Interobserver agreement: Cohen’s weighted kappa value score $s$

	Static	BG-MC	DDG-MC
N1	0.88	0.93	0.92
N2	0.92	0.95	0.94
N3	0.91	0.91	0.87
M1	0.96	0.94	0.95

### Influence of motion correction on assessment of lymph nodes and distant metastases

**Fig. 2:**
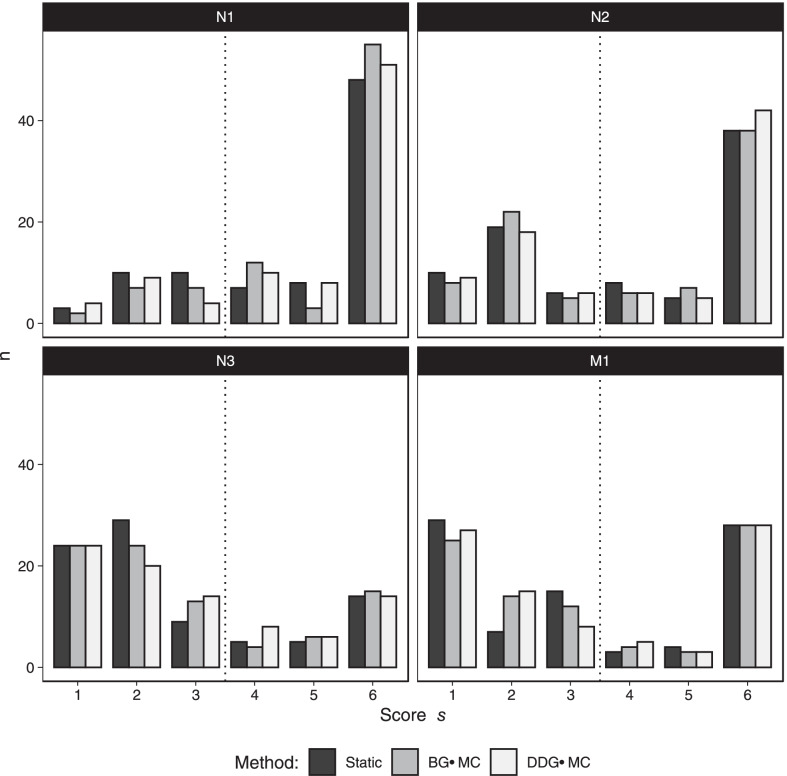
Distribution of score $s$ for N1 to N3 and M1 by image reconstruction method summed for both readers (N = 2*43 = 86)

The mean scores $s$ for reader 1 and lymph node region N1 were 4.79, 5.00 and 4.95 for static, BG-MC and DDG-MC images, respectively. For the other lymph node regions and M1, the following mean scores were observed for static, BG-MC, and DDG-MC: 4.14, 4.16, 4.30 for N2, 2.77, 2.93, 2.91 for N3, and 3.30, 3.33, 3.28 for M1, respectively. For reader 2 the mean scores for static, BG-MC and DDG-MC images were as follows: 4.72, 5.00, 4.81 for N1, 4.02, 4.07, 4.16 for N2, 2.77, 2.81, 2.95 for N3, and 3.40, 3.37, 3.33 for M1, respectively. Mean and median scores for both readers combined are given in Table 3. Differences in scoring between image reconstruction methods are visualized in Fig. 2.

**Table 3:** Mean and median score $s$ for N1, N2 and N3 and different methods.

	Static	BG-MC	DDG-MC	*p* value
*N1*				
Mean	4.76 (1.64)	5.00 (1.50)	4.88 (1.61)	0.004*
Median	6.00 [3.00]	6.00 [2.00]	6.00 [2.00]	
*N2*				
Mean	4.08 (1.97)	4.12 (1.95)	4.23 (1.96)	0.036**
Median	4.50 [4.00]	5.00 [4.00]	5.00 [4.00]	
*N3*				
Mean	2.77 (1.79)	2.87 (1.82)	2.93 (1.79)	0.295
Median	2.00 [3.00]	2.00 [3.00]	2.00 [3.00]	
*M1*				
Mean	3.35 (2.12)	3.35 (2.09)	3.30 (2.13)	0.850
Median	3.00 [5.00]	3.00 [5.00]	3.00 [5.00]	

Standard deviation in round parentheses, interquartile range in square brackets

^*^ *p* values of post hoc test: 0.001, 0.026 and 0.122 for BG-MC versus static, BG-MC versus DDG-MC and static versus DDG-MC

^**^ *p* values of post hoc test: 0.676, 0.042 and 0.016 for BG-MC versus static, BG-MC versus DDG-MC and static versus DDG-MC

Analyzing the data of both readers revealed statistically notable differences in score $s$ between the reconstruction methods for lymph node regions N1 and N2 (*p* = 0.004 and *p* = 0.036, Table 3). For N1, BG-MC images showed a significantly higher score compared to static and DDG-MC images (*p* = 0.001 and 0.026), whereas no notable difference was evident between static and DDG-MC images (*p* = 0.122). For N2, DDG-MC images showed a significantly higher score compared to static and BG-MC (*p* = 0.016 and 0.042), whereas no notable difference was evident between static and BG-MC images (*p* = 0.676) (Table 3).

For the dichotomized score $d$, there were several cases where motion correction with either BG or DDG resulted in uprating consensually for both readers. However, there was no case in which both readers rated down any station in motion-corrected images compared to static images. Compared to static images there where three cases where both readers rated up N1 from tumor-free to metastatic (Table 4). For DDG-MC there were two cases where both readers rated up N1 and one case where both readers rated up station N2 (Table 4).

**Table 4:** Up- or downrating in consensus by the readers between different image reconstruction methods

	Method		
	BG-MC vs. Static	DDG-MC vs. Static	BG-MC vs. DDG-MC
*N1*			
Same	40	41	42
Up	3	2	0
Down	0	0	1
*N2*			
Same	43	42	42
Up	0	1	1
Down	0	0	0
*N3*			
Same	43	43	43
Up	0	0	0
Down	0	0	0
*M1*			
Same	43	43	43
Up	0	0	0
Down	0	0	0

**Fig. 3:**
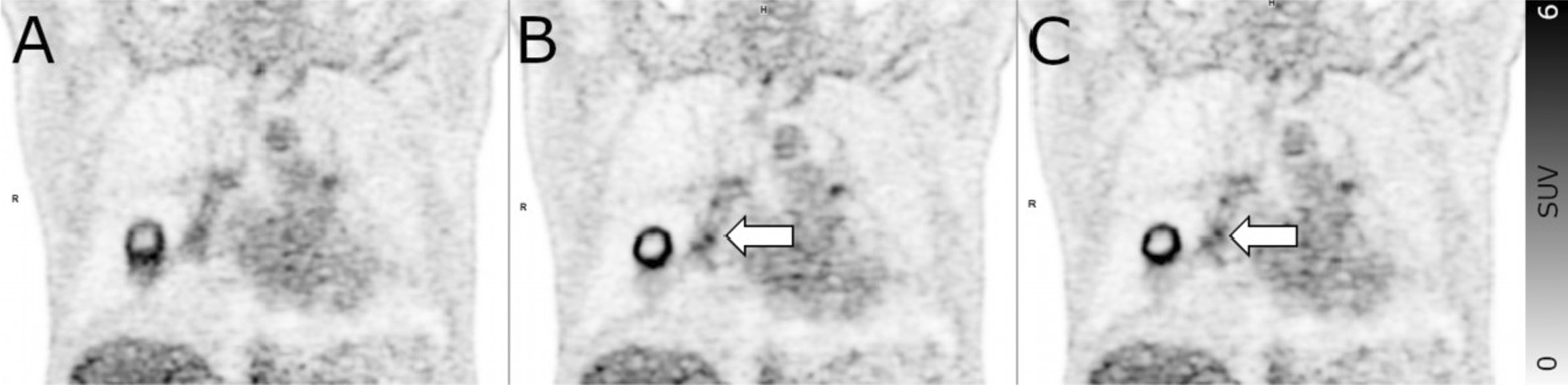
Coronal PET images of a patient with non-small cell lung cancer in the right lung. Both readers rated N1 as free of metastases on static images (**A**). In contrast to the static images, areas of focal tracer accumulation are discernable in the right lung hilus on both BG-MC (**B**) and DDG-MC (**C**) images (arrows). Both readers rated N1 as metastatic on BG-MC and DDG-MC images. Transbronchial needle aspiration confirmed the presence of metastases in ipsilateral and contralateral mediastinal lymph nodes

Correlative histopathological results from multisegmental EBUS-TBNA were available for one patient in whom both BG- and DDG-based motion correction resulted in uprating of N1 from tumor free to metastatic and DDG-based motion correction resulted in uprating of N2 from tumor free to metastatic. EBUS-TBNA results confirmed metastasis in ipsilateral and contralateral lymph nodes (Fig. 3).

### Influence of motion correction on certainty scores

**Fig. 4:**
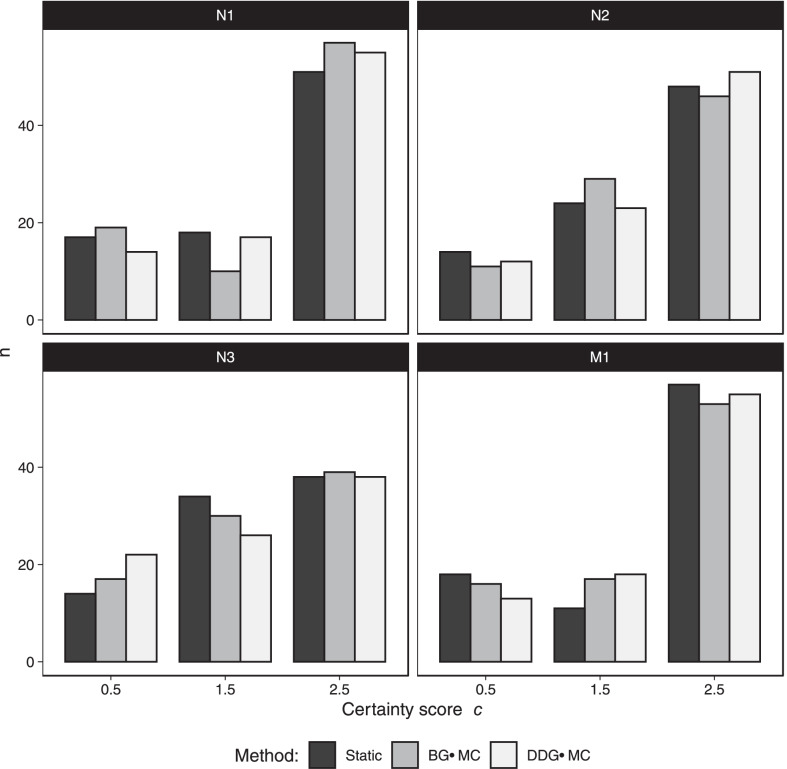
Distribution of certainty $c$ for N1 to N3 by image reconstruction method summed for both readers (N = 2*43 = 86)

No notable differences in the certainty scores $c$ were found between the reconstruction methods (Table 5 and Fig. 4).

**Table 5:** Mean and median certainty score $c$ for different reconstruction methods.

	Static	BG-MC	DDG-MC	*p* value
*N1*				
Mean	1.90 (0.80)	1.94 (0.83)	1.98 (0.76)	0.28
Median	2.50 [1.00]	2.50 [1.00]	2.50 [1.00]	
*N2*				
Mean	1.90 (0.76)	1.91 (0.71)	1.95 (0.73)	0.41
Median	2.50 [1.00]	2.50 [1.00]	2.50 [1.00]	
*N3*				
Mean	1.78 (0.73)	1.76 (0.77)	1.69 (0.82)	0.57
Median	1.50 [1.00]	1.50 [1.00]	1.50 [1.75]	
*M1*				
Mean	1.95 (0.82)	1.93 (0.79)	1.99 (0.75)	0.80
Median	2.50 [1.00]	2.50 [1.00]	2.50 [1.00]	

Standard deviation in round parentheses, interquartile range in square brackets

### Influence of motion correction on SUV and metabolic tumor volume

Histogram analysis revealed non-normal distributions for SUV and MTV values (*p* < 0.05 in Shapiro–Wilk tests). Differences were evident between image reconstruction methods for SUV_max_, SUV_mean_ and MTV for all lymph node regions and for the primary tumor (all p values for Friedman’s test < 0.001, Table 6). Post hoc testing demonstrated significantly higher SUV_max_ and SUV_mean_ and smaller MTV for BG-MC and DDG-MC images compared to static images for all locations (all *p* values < 0.003). No significant differences for SUV or MTV were found between BG-MC and DDG-MC.

**Table 6:** Median SUV and MTV for different methods and location, interquartile range in square brackets

	Static	Belt	DDG	*p* value
*Primary*				
SUVmax	16.6 [16.2]	20.2 [16.2]	20.5 [13.4]	< 0.001
SUVmean	9.9 [8.6]	11.2 [7.9]	11.2 [7.6]	< 0.001
MTV	5.1 [15.2]	4.2 [14.4]	4.0 [14.2]	< 0.001
*N1*				
SUVmax	7.4 [9.2]	9.6 [9.7]	9.8 [11.2]	< 0.001
SUVmean	4.2 [5.8]	5.2 [6.7]	5.4 [6.8]	< 0.001
MTV	4.0 [5.4]	2.6 [4.1]	2.4 [4.3]	< 0.001
*N2*				
SUVmax	8.1 [9.2]	8.7 [11.0]	8.6 [10.9]	< 0.001
SUVmean	4.3 [5.4]	4.9 [6.5]	4.5 [6.2]	< 0.001
MTV	3.0 [4.1]	2.4 [3.2]	2.8 [3.2]	< 0.001
*N3*				
SUVmax	6.3 [5.3]	7.5 [7.1]	7.3 [6.8]	< 0.001
SUVmean	3.4 [3.3]	4.1 [4.5]	4.0 [4.7	< 0.001
MTV	6.1 [5.5]	3.9 [3.4]	3.7 [2.5]	< 0.001

## Discussion

State-of-the-art staging of lung cancer patients often includes initial staging with F-18-FDG PET/CT, especially for the assessment of lymph nodes and distant metastases following the updated 8th edition of TNM classification [26]. Clinically available hardware-based gating (in our case, belt-based gating) and DDG are promising methods to overcome PET inherent disadvantages in the assessment of lesions affected by respiratory motion [10, 17]. Besides the well-known advantages of motion-corrected PET, i.e., higher, more accurate tracer uptake values and subjectively “sharper” images, studies on the impact of motion-corrected PET on staging and value in clinical decision-making are still sparse [10, 21, 24, 30, 31]. This study therefore sought to evaluate the impact of two different methods of fully motion-corrected PET reconstructions compared to standard static (non-motion-corrected) PET on lung cancer staging scans.

In line with previous studies, semi-quantitative PET uptake values SUV_max_ and SUV_mean_ were significantly higher in primary tumor and metastatic lesions in our study (Table 6) when applying motion correction [21, 24, 31]. SUV was not significantly different for BG-MC and DDG-MC in the presented study in line with previously published results based on the same methodology [21]. Contrary to these results, Walker et al. reported only slightly but significantly higher SUV for DDG compared to external device-based gating in 144 patients; however, both of their gating methods are different than the ones employed by us [32]. More specifically, their applied hardware-based gating method relies on camera tracking of body surface markers, and their DDG algorithm uses principal component analysis rather than spectral Fourier analysis as in our case. Furthermore, a different patient collective was analyzed, making a direct comparison between their results and ours difficult. However, they mention that their camera-based gating approach relied on a prospective trigger insertion algorithm into the list mode stream rather than a retrospective one they used for DDG. This might explain a perceived superiority of their DDG, while in our case both gating approaches relied on a retrospective analysis of the acquired waveforms, thus explaining very similar SUV for both motion-corrected PET images.

In line with previously published results, MTV was significantly smaller when applying gating methods compared to static PET [30, 33, 34]. This is of utmost importance for target volume delineation in radiotherapy planning, not only limited to lung cancer treatment, although the clinical impact of these changes still warrants further investigation [35].

We theorized that the effect of PET motion correction, i.e., increasing SUV while decreasing lesion volumes at the same time, could result in human readers perceiving lesions as showing focal tracer uptake compatible with malignant lesions which would have been rated as benign or even overlooked on static images (Fig. 3). Going beyond most previous studies, our study could indeed demonstrate that motion-corrected PET does not only result in higher SUV and smaller MTV but may also impact staging decision by human readers, even if only in a limited amount of cases. On average, motion correction with BG-MC and DDG-MC made readers assign significantly higher scores compared to static images for lymph nodes in N1 and N2 but not in N3. Therefore, the readers were more likely to classify lymph nodes in N1 (for BG-MG) and N2 (for DDG-MC) as metastatic compared to static images. The reason why classification of N1 and N2 but not N3 and M1 are affected by PET motion correction might be related to the fact that lymph nodes in N1 and N2 are more affected by respiratory motion than those in N3 which can have a larger distance to the diaphragm, e.g., in the case of cervical lymph node metastases. Moreover, M1 does not only include patients with a single metastasis potentially affected by respiratory motion as in the adrenal gland or the liver, but also patients with (additional) multiple bone metastases not or barely affected by respiratory motion.

On average, the certainty score $c$ of the readers was not different between the reconstruction types. We believe this is connected to the observed shift in $s$ to higher values over the whole range of possible outcomes; thus, cases that were ambiguous without motion correction had the tendency to be perceived as metastases with motion correction, while motion correction may also lead to lymph nodes being classified as potential metastases that were deemed unsuspicious without motion correction.

Following the application of motion correction, uprating from disease free to metastatic on the dichotomous score occurred, consensually for both readers, in 3/43 (7%) patients in N1 using BG-MC and in 2/43 (5%) patients using DDG-MC. For N2, consensual upstaging occurred in 1/43 patient with DDG-MC (2%). Correlative histopathological results from multisegmental EBUS-TBNA were available for one patient confirming uprating of both N1 and N2 with DDG-MC as true positive. This underlines the clinical impact of our findings.

Migration of lymph node disease status, seen with PET motion correction in this study, could thus have potentially resulted in a change in clinical patient management. Uprating of lymph nodes in N2 in one case could have shifted primary treatment from surgery to definitive chemoradiotherapy. Migration of disease status of N1 in three cases could have affected further workup, as new ESMO guidelines recommend EBUS-TBNA for mediastinal lymph nodes only with additional risk factors such as cN1 [36].

Our results corroborate the findings of previous studies investigating the impact of motion correction on lesion detectability and clinical management: In a study by Sigfridsson et al., comprising 7 patients with liver metastases, DDG resulted in the detection of 41 liver lesions compared to 36 lesions with static image reconstruction [31]. In a mixed cohort of 149 patients with different tracers (i.e., FDG, PSMA and DOTATATE) and underlying pathologies included, Messerli et al. detected a higher number of metastases with DDG in organs affected by respiratory motion in up to 27% of patients included [24, 31]. A higher number of lesions does not automatically result in change in clinical stage or management [36]. Nevertheless, Messerli et al. demonstrated a change in clinical management in 8% of patients in their cohort, corroborating our result that gating or motion correction can result in a change in clinical management [24]. In the only other dedicated study on *n* = 55 lung cancer patients, relying on 7th edition of the TNM classification, T and M staging remained unchanged when applying hardware-based respiratory gating and changes in N stage occurred in 7% or 13% depending on the reader [37]. These results are in line with the results of our study for BG-MC and DDG-MC. Besides relying on 7th edition of the TNM classification the gating approach used in the study by Grootjans et al. is significantly different from ours, since only belt-driven gated and not fully motion-corrected PET was investigated.

One of the main limitations of our study is inherent to clinical reader assessment, as readers cannot be completely blinded to the image appearance of different reconstruction images. However, by using two different methods of gating this disadvantage might be less applicable in this study than in others with only one method of gating [24]. By applying an interval of at least two weeks between reading the different datasets and by mixing different patients and reconstruction methods in one session bias is reduced. Consecutive patients were retrospectively included, and we thus had no influence on clinical stage of the patients at initial diagnosis. As previously reported gating has only a limited impact in advanced tumor stages [37]. Histopathological correlation was established for uprating N1 and N2 in one patient where dedicated EBUS-TBNA biopsy of different lymph node stations was available. In this study we included the most commonly used methods of gathering respiratory data from patients, hardware/belt-based assessment of motion and DDG and used them as a basis for full elastic motion correction; thus, a direct comparison of our results to studies using less complex gating methods alone is challenging.

To conclude, this pilot study offers first insights into the clinical impact of motion correction for F-18-FDG PET on staging scans of lung cancer patients following the 8th edition of TNM classification. Full motion correction using hardware-based and data-driven gating both seem to have a similar clinical impact on uprating in few patients with limited disease while significantly influencing quantitative PET uptake parameters.

## Data Availability

All data generated or analyzed during this study can be provided upon reasonable request.

## Abbreviations

F-18-FDG: Fluorodeoxyglucose
BG: Belt gating
CT: Computed tomography
DDG: Data-driven gating
EBUS-TBNA: Endobronchial ultrasound-guided transbronchial needle aspiration
PET: Positron emission tomography
MTV: Metabolic tumor volume
SUV: Standardized uptake value
